# The multitargeted receptor tyrosine kinase inhibitor sunitinib induces resistance of HER2 positive breast cancer cells to trastuzumab-mediated ADCC

**DOI:** 10.1007/s00262-022-03146-z

**Published:** 2022-01-23

**Authors:** Eliza Guti, Zsolt Regdon, Isotta Sturniolo, Alexandra Kiss, Katalin Kovács, Máté Demény, Árpád Szöőr, György Vereb, János Szöllősi, Csaba Hegedűs, Zsuzsanna Polgár, László Virág

**Affiliations:** 1 Department of Medical Chemistry, Faculty of Medicine, University of Debrecen, Debrecen, Hungary; 2 Doctoral School of Molecular Medicine, University of Debrecen, Debrecen, Hungary; 3 MTA-DE Cell Biology and Signaling Research Group, Debrecen, Hungary; 4 Department of Biophysics and Cell Biology, Faculty of Medicine, University of Debrecen, Debrecen, Hungary

**Keywords:** Sunitinib, Natural killer cell, Breast cancer, ADCC, Trastuzumab, Herceptin

## Abstract

Despite recent advances in the development of novel personalized therapies, breast cancer continues to challenge physicians with resistance to various advanced therapies. The anticancer action of the anti-HER2 antibody, trastuzumab, involves antibody-dependent cell-mediated cytotoxicity (ADCC) by natural killer (NK) cells. Here, we report a repurposing screen of 774 clinically used compounds on NK-cell + trastuzumab-induced killing of JIMT-1 breast cancer cells. Using a calcein-based high-content screening (HCS) assay for the image-based quantitation of ADCC that we have developed and optimized for this purpose, we have found that the multitargeted tyrosine kinase inhibitor sunitinib inhibits ADCC in this model. The cytoprotective effect of sunitinib was also confirmed with two other assays (lactate dehydrogenase release, and electric cell substrate impedance sensing, ECIS). The drug suppressed NK cell activation as indicated by reduced granzyme B deposition on to the target cells and inhibition of interferon-γ production by the NK cells. Moreover, sunitinib induced downregulation of HER2 on the target cells' surface, changed the morphology and increased adherence of the target cells. Moreover, sunitinib also triggered the autophagy pathway (speckled LC3b) as an additional potential underlying mechanism of the cytoprotective effect of the drug. Sunitinib-induced ADCC resistance has been confirmed in a 3D tumor model revealing the prevention of apoptotic cell death (Annexin V staining) in JIMT-1 spheroids co-incubated with NK cells and trastuzumab. In summary, our HCS assay may be suitable for the facile identification of ADCC boosting compounds. Our data urge caution concerning potential combinations of ADCC-based immunotherapies and sunitinib.

**Supplementary Information:**

The online version contains supplementary material available at 10.1007/s00262-022-03146-z.

## Introduction

Although a slight overall decrease in European cancer mortality has recently been reported, cancer statistics still show a grim picture [1]. Based on data from the Eurostat and WHO, it is estimated that the number of cancer-related deaths in the EU in 2021 is expected to be 1.2 million (670,338 in men and 524,985 in woman). Although showing a declining trend, breast cancer is still the most common type of cancer in woman claiming more than 80,000 lives each year in the European Union only [1]. Treatment options available for breast cancer patients include surgery, combination chemotherapy, radiation therapy, hormone therapy (for estrogen or progesterone receptor positive cases), anti-HER2 antibodies (e.g., trastuzumab) and PARP inhibitors (for tumors with germline mutations in the BRCA gene). Moreover, a promising novel approach is represented by immune checkpoint inhibitors (e.g., anti-PDL1 antibodies) which have recently been added to the therapeutic weaponry for the fight against breast cancer. However, despite these numerous therapeutic options, the treatment of breast cancer and triple negative breast cancer in particular poses a real challenge for oncologists. To overcome resistance to traditional therapeutic regimes, novel combinations of the above therapies are often tested both in preclinical models and in various clinical trials. Furthermore, a number of cell therapies (e.g., with immune vaccines or chimeric antigen receptor expressing T cells, (CAR T cells)) have been proposed to be potential new treatment modalities in the future.

Natural killer (NK) cells belong to the family of innate cytotoxic lymphocytes [2]. Whereas cytotoxic T cells are effective against tumors that present MHC class I-associated tumor antigen peptides, NK cells work effectively when cancer cells downregulate MHC proteins. The activation state of NK cells is determined by a balance between stimulation of activating and inhibitory receptors [3]. For example, MHC I expression by the tumor cells is sensed by NK cell inhibitory KIR receptors [4]. Natural cytotoxicity receptors, on the other hand, function independently of MHC proteins and sense molecular patterns associated with tumor transformation or stress [5]. Various other NK cell surface receptors such as immunoglobulin like transcripts or C-type lectin receptors also contribute to shaping the activation state of NK cells.

Anti-HER2 antibodies (e.g., trastuzumab) represent an efficient treatment modality for HER2 positive breast cancer and potentially other types of HER2 positive cancers. Interestingly, trastuzumab can reduce the number of circulating and disseminating cancer cells even if the primary tumor is resistant to trastuzumab [6, 7]. Expression of Fc receptors on NK cells is considered crucial for an efficient treatment with trastuzumab because NK cells partner with antibodies for cancer cell killing by antibody-dependent cell-mediated cytotoxicity (ADCC) [8–10].

It appears that understanding mechanisms regulating the sensitivity of cancer cells to anti-growth factor receptor antibody therapy with special regard to ADCC may be crucial for the development of novel, more efficient treatment modalities. Moreover, combination therapies involving virtually all types of traditional, immune or cell therapies have been tested extensively to identify synergizing combinations. Therefore, we set out to develop a new high-content analysis assay for the quantification of trastuzumab-mediated ADCC. By screening a small compound library, we identified the multitargeted tyrosine kinase inhibitor sunitinib used for the treatment of various types of cancer, as a compound inducing resistance to trastuzumab-dependent ADCC in JIMT-1 breast carcinoma cells. Follow-up investigations revealed that on the one hand, sunitinib impairs the killing function of NK cells, while on the other hand, it induces autophagy, downregulation of HER2 expression and enhances surface attachment of JIMT-1 cells, potential mechanisms underlying the desensitizing effect of the compound in trastuzumab-dependent ADCC.

## Materials and methods

### Cell lines

JIMT-1 breast cancer cells were cultured in DMEM/F-12 medium (Sigma, D8437) supplemented with 20% fetal bovine serum (FBS, Biosera, FB-1090/500), 0.3 U/ml insulin (100 NE/ml, Humulin R, HI0219), and 1% penicillin–streptomycin (Biosera, LM-A4118/100). The human breast cancer cell lines (SKBR3, MDA-MB468-HER2) and the human gastric adenocarcinoma cell line (MKN7) were cultivated in high glucose DMEM medium (LM-D1111, Biosera) supplemented with 10% FBS and 1% penicillin–streptomycin. CD16.176 V.NK-92 cells were supplied by Dr. Kerry S. Campbell, (the Fox Chase Cancer Center, Philadelphia, PA on behalf of Brink Biologics, Inc. San Diego, CA) and were generated from the NK-92 cell line (ATCC CRL-2407) derived from a human NK-like phenotype non-Hodgkin’s lymphoma, which lacks endogenous expression of CD16 (Gong, Maki, et al. 1994). This has been transduced to express a high affinity variant (176 V) of FcγRIIIA (CD16, see VAR_003960 entry within P08637 and BC017865.1 [11, 12]).

The NK cells were maintained in α-MEM (Sigma, M8042) supplemented with 20% FBS, 1% MEM-NEAA (Gibco, 11,140–050), 1% Na-pyruvate (Lonza, BE13-115E), 1% glutamine (Gibco, 35,050–061), 1% penicillin–streptomycin and 100 IU/ml IL-2 (Proleukin, Novartis Hungária Kft., Budapest, Hungary, PHC0026). All cell lines were cultured in a humidified atmosphere containing 5% CO_2_ at 37 °C and were routinely checked for the absence of mycoplasma contamination.

### Electric-cell substrate impedance sensing (ECIS) measurement

JIMT-1 cells were seeded in 8-well chambers (Ibidi, 8W10E) after coating with JIMT-1 medium for 1 h. 10^5^ cells formed a confluent monolayer on the electrodes. The effector/target ratio was 2:1 which was determined during the optimization phase of the assay. After the impedance of the target JIMT-1 cells has reached a plateau (24 h), the effector cells were added together with trastuzumab antibody (humanized anti-HER2 monoclonal antibody, 10 µg/ml, Herzuma®, EGIS Pharmaceuticals, Budapest, Hungary). Equal volume of medium was pipetted into control wells. The impedance was measured at 4000 Hz at 4 s intervals. Impedance values were normalized to the value of the same well measured before the treatment. The chambers were kept in an incubator with humidified atmosphere containing 5% CO_2_ at 37 °C.

### High-content screening (HCS) assay for the identification of ADCC modifying drugs

The outline of the HCS assay is presented on Suppl. Fig. S1. Briefly, the 96-well HCS plates (Cell Carrier Ultra HCS microplates, PerkinElmer, Waltham, MA, USA) were pre-coated with JIMT-1 medium. JIMT-1 cells (10 000/well) were added in a volume of 75 µl and 24 h later, target cells were stained with 0.5 μM calcein-AM (Sigma, 177,783) for 1 h at 37 °C. After washing with medium, wells were covered with 50 μl medium and cells were then treated for 1 h with a compound library (20 µM, Enzo, Screen-Well® FDA Approved Drug Library V2) using a Freedom EVO liquid handling robot (TECAN, Switzerland). By adding 20,000 unstained NK effector cells (E:T = 2:1) and trastuzumab antibody (10 µg/ml, diluted in JIMT-1 media) or the chimeric (mouse/human) cetuximab antibody (anti-EGFR antibody, 2 µg/ml, Erbitux®, Merck, Darmstadt, Germany) to each well, the co-culture was incubated for 3 h. (Final concentration of test compounds was 10 µM). Images were taken with an Opera Phenix High-Content Analysis equipment (PerkinElmer, Waltham, MA, USA) at the beginning (0 h) and at the end (3 h) of ADCC using 10 × air objective with 0.3 numerical aperture in non-confocal mode (ex 488 nm; em 500–550 nm channel). The extent of ADCC efficiency was determined with the Harmony software (Perkin Elmer). The adherent calcein-stained JIMT-1 cells were counted with the “Find cells” software module (channel: calcein, method: B, common threshold: 0.39, area: > 600 µm^2^, splitting coefficient: 20.4, individual threshold: 0.16, contrast: > 0.04). The SKBR3 and MDA-MB468-HER2 cells were detected with the M method, diameter: 40 µm, splitting sensitivity: 0.20, common threshold: 0.20. The MKN7 cells were found in M method, diameter: 40 µm, splitting sensitivity: 0.70, common threshold: 0.19.

The percentage of surviving target cells was calculated with the following equation:

$${\text{Cell\;survival}}\; (\% ) = 100 {\text{(target\;number}}_{\text{3h}}/{\text{target\; number}}_{\text{0h}})$$

The drugs which caused at least 20% change in cell survival were considered as hits.

### LDH assay

Target cells (4000/well) were seeded into 96-well plates. On the next day, the target cells were pre-treated with the test compounds for 1 h. Effector cells (8000/well) were added in trastuzumab-containing (10 µg/ml) JIMT-1 medium (5% FBS), and the co-culture was incubated for 6 h. After centrifuging the plate (250 g, 4 min), the supernatant was removed and mixed in a 1:1 ratio with the LDH reagent (Invitrogen-Thermo Scientific, Waltham, MA, USA Cat#C20301) according to the kit’s manual. The absorbance was detected with a Multiskan™ FC Microplate Photometer (Thermo Scientific, Waltham, MA, USA).

Cytotoxicity was calculated with the following formula:

$${\text{Cytotoxicity }}\left[ \% \right] = {1}00 \times \left( {{\text{OD}}_{{{\text{E}} + {\text{T}} + {\text{A}}}} - {\text{OD}}_{{{\text{E}} + {\text{T}}}} } \right)/\left( {{\text{OD}}_{{\text{T lysed}}} - {\text{OD}}_{{\text{T}}} } \right)$$

where E represents effector cells, T represents target cells and A indicates the presence of trastuzumab antibody.

### Characterization of cell morphology

JIMT-1 cells seeded in 96-well Cell Carrier Ultra HCS microplates (4000/well) were stained in JIMT-1 medium with DRAQ5 fluorescent dye (5 µM dissolved in JIMT-1 medium, 30 min, 37 °C), and the cells were analyzed using an Opera Phenix High-Content Analysis equipment. The images were taken with a 20 × objective (0.4 numerical aperture) in confocal mode. Morphometric analysis was performed with the Harmony software. First, the cell numbers were detected with the “Find nuclei” option (channel: Alexa 633, method: B, common threshold: 0.15, area: > 50 µm^2^, splitting coefficient: 11.6, individual threshold: 0.35, contrast: > 0.35). Then, the cell borders were found using the “Find cytoplasm” building block (channel: Alexa 633, method: A, individual threshold: 0.10). In the following step, in the “Select Population” mode (population: nuclei, method: common filters), the morphology of nuclei was characterized with the “Calculate Morphology Properties [1, 2 and 3]” option (population: nuclei selected [1–3], region: nucleus [1], cytoplasm [2] or cell [3], method: standard [1–3]).

### Dispase assay

Cell adhesion was determined as previously described [13] with modifications as follows. Target cells (60,000/well) were seeded into 24-well plates. Target cells were pre-treated with 10 µM sunitinib malate (PZ0012) dissolved in DMSO or 0.1% DMSO as control. Plates were incubated for 3 h. After removing the supernatant, cells were washed with ice-cold PBS (Lonza, BE17-517Q) and 200 µl 0.6 U/ml dispase (Gibco, 17,105–041) was added. Plates were then incubated for 35 min at 37 °C. The enzyme was aspirated and the attached cells were washed 5 × with 100 µl PBS. Subsequently, the cells were fixed in 10% TCA (09,290–101-190, Molar Chemicals Ltd., Halásztelek, Hungary) overnight at 4 °C. On the next day, plates were washed once with PBS, dried and stained with 0.4% (m/v) sulforhodamine b (Sigma, #230,162) dye for 10 min at room temperature. The cells were washed with 1% acetic acid, dried and the bound dye was dissolved in 1 mM Tris base. The absorbance was detected at 515 nm with a Tecan Spark multimode microplate reader.

### Intracellular granzyme B staining

Target cells (10,000/well) were seeded in 96-well plates. Subsequently, the attached cells were stained with 5 µM Cell Tracker Blue dye (Invitrogen-Thermo Scientific, Waltham, MA, USA, C2110) in DMEM/F12 media for 30 min at 37 °C. Cells were then washed twice with DMEM/F12 medium and pre-treated with sunitinib malate for 1 h. ADCC was started by the addition of the NK cells (20,000/well) and trastuzumab (10 µg/ml). The final concentration of sunitinib was 10 µM. The co-culture was incubated for 3 h, and then, the cells were collected and washed with PBS. Cells were then fixed in 4% formalin (10 min, RT) and permeabilized with 0.1% Triton X-100 solution (T8787, Sigma, 10 min, RT). Samples were blocked in 1% BSA for 1 h and incubated with anti-granzyme B Alexa Fluor 647 antibody (Biolegend, San Diego, CA, cat#515,405, 200 × dilution) for 20 min at room temperatures in dark. The number of granzyme B positive cells were determined by flow cytometry using a NovoCyte flow cytometer.

### Cytokine secretion assay

2 × 10^5^ CD16.176 V.NK-92 cells (in the presence or absence of 10 µg/ml trastuzumab) were plated onto plates pre-coated with 1 µg/ml HER2-Fc (R&D Systems, Minneapolis, MN, USA) in complete NK cell medium supplemented with 0.01–30 µM Sunitinib. Following 24 h of culture, supernatant was harvested and analyzed for the presence of interferon-gamma (IFNγ) by ELISA (R&D systems, Minneapolis, MN, USA) according to the manufacturer's instruction using a Spark® Multimode Microplate Reader (Tecan, Switzerland).

### Cell cycle analysis

Cells were trypsinized and then fixed in ice-cold 70% ethanol at 4 °C for 30 min. After washing cells with PBS, samples were treated with 1 mg/ml RNase (Sigma, R6513) for 30 min at 37 °C and then stained with 5 µM DRAQ5 (eBioscience-Thermo Fisher Scientific, Waltham, MA, USA; Cat#65-0880-96) for 30 min at room temperature. The DNA content was measured with a NovoCyte flow cytometer.

### Western blot

The cells were washed once in PBS and collected by scraping into 150 µl of RIPA buffer (50 mM Tris–HCl, 1% Igepal, 0.25% Na-deoxycholate, 150 mM NaCl, 1 mM EDTA, pH 7.4) supplemented with 1% PMSF (phenylmethylsulfonyl fluoride, Sigma P7626) and 1% protease inhibitor cocktail (Sigma P8340). The extracts were sonicated, and the supernatants were collected after centrifugation. The protein concentration was determined with a Direct Detect Spectrometer. Samples were diluted in SDS sample buffer (0.31 M Tris–HCl, pH 6.8, 50% glycerol, 10% SDS, 100 mM DTT, 0.01% bromophenol blue, 1 M β-mercaptoethanol (Applichem Gmbh, #A1108,0100) and then boiled at 95 °C for 10 min. 20 µg protein was separated in 12% SDS-PAGE and transferred to nitrocellulose membranes. The membranes were blocked in 5% non-fat dry milk in Tris-buffered saline-Tween20 (TBS; 25 mM Tris, 0.136 M NaCl, 2.682 mM KCl; 0.1% Tween20, pH 7.4) for 60 min. The primary antibodies were diluted in 1% non-fat dry milk, and the membranes were incubated overnight at 4 °C. On the next day, the membranes were washed with TBS 0.1% Tween20 (3 times for 10 min), and then, the HRP-conjugated secondary antibody was added in 1% non-fat dry milk for 1 h at room temperature. After washing membranes with TBS 0.1% Tween20 (3 times for 10 min) ECL-Pico reagent (Thermo Scientific, #34,580) was added for HER2, Akt, pAkt, and β-actin, 10 × diluted ECL-Femto reagent (#34,094, Thermo Scientific) for LC3B. For the detection of Akt, the pAkt antibody was removed by mild stripping (stripping buffer: 15 g glycine, 1 g SDS, 10 ml Tween20 in 1 l dH_2_O, pH 2.2) for 8 min. Blots were imaged with a Chemidoc Imaging system (Bio-Rad Hungary Ltd, Budapest, Hungary) and were quantitated with ImageJ software.

**Table:** 

	Antibody specific for	Host species	Cat#	Dilution	Supplier
Primary antibodies	LC3B	rabbit	NB100-2220	5000x	Novus Biologicals
	β-actin	mouse	sc-47778	3000x	Santa Cruz Biotechnology
	HER2	mouse	OP-15	500x	Sigma-Aldrich
	pAkt (S473)	rabbit	4058S	1000x	Cell Signaling
	Akt	rabbit	4691S	1000x	Cell Signaling
Secondary antibodies	Anti-rabbit IgG, HRP-linked	goat	7074S	2000x	Cell Signaling
	Anti-mouse IgG, HRP-linked	horse	7076S	2000x	Cell Signaling

### Generation of JIMT-1 cells expressing Enhanced Green Fluorescent protein (EGFP)

For lentivirus production, HEK293 cells were plated the day before the procedure and allowed to grow to 80–90% confluence. Cells were transfected with the following four plasmids—pLP-1, pLP-2, pLP-VSV-G (Invitrogen) and pWOX-EGFP (a kind gift from Prof. József Tőzsér, University of Debrecen) 10 µg total, in a 1:1:1:1 ratio using Lipofectamine 3000. The medium was replaced 24 h later and virus containing medium was collected 48 h after the transfection. The medium was filtered through a 0.45 μm syringe filter and added to JIMT-1 cells together with 8 μg/ml polybrene. Five days after viral transduction, cells expressing high level of EGFP were collected with a FACSAria cell sorter, expanded into culture flasks, grown and frozen in aliquots.

#### High content analysis of cell death in spheroids

JIMT-1-EGFP (Enhanced Green Fluorescent Protein) cells were used for the generation of spheroids. Cells were grown in 96-well plates pre-coated with 0.5% agarose-PBS solution to form a U-shaped, cell-repellent bottom. Cells were seeded onto plates at 2 × 10^4^/ml seeding density in 100 μl cell suspension per well. The cells were allowed to clump together, and size and shape of spheroids were regularly checked. On day 3, spheroids were transferred to glass bottom Cell Carrier-96 ultra microplates (PerkinElmer, Waltham, MA, USA) that were previously coated with Pluronic-F127 (0.5% in DMSO, Sigma #P2443, 45 min at room temperature) in triplicates. NK cells (E:T ratio of 20:1) and trastuzumab (10 µg/ml) were then added to the wells in the absence or presence of sunitinib (20 μM). Specifically, spheroids were pre-treated with sunitinib for 1 h and ADCC was started with the addition of NK cells (prestained with 10 µM Cell Tracker Blue for 1 h) and trastuzumab as previously described. After 24 h, the cells were stained with Annexin V-Alexa Fluor™ 647 conjugate (A23204, Invitrogen-ThermoFisher Scientific) for 1 h in growth medium to measure cell death. Spheroid samples were imaged with Perkin Elmer Opera Phenix High-Content Analyzer (10 × objective with 0.3 numerical aperture in confocal mode) at the following channels: EGFP (ex: 488 nm em: 510 nm), Alexa647 (ex: 640 nm, em: 650–760 nm). Images were analyzed with the Harmony software (Perkin Elmer). Spheroids were identified by the EGFP fluorescence of JIMT-1 cells by using “Find texture region” option, and filtered out by size (> 25,000). Border objects were removed by the “Select population” option. Annexin positive (apoptotic) cells appeared on the periphery of spheroids. Therefore, Annexin intensity was measured in this apoptotic “ring”, selected by the “Select region” option (outer border −90%). Annexin intensities were determined and expressed as mean intensity.

### Intracellular LC3B staining

After treatments, JIMT-1 cells (10 000/well) were washed with PBS and then fixed in -20 °C methanol for 30 min. After the fixation, the cells were washed and permeabilized with 0.1% Triton X-100 for 10 min at room temperature. The cells were then blocked in 1% BSA for 1 h at room temperature. Anti-LC3B primary antibody (500 × in 1% BSA; NB100-2220, Novus Biologicals) was added and samples were incubated overnight at 4 °C. On the next day, the cells were washed with PBS, stained with DAPI (1 µg/ml, A1001, PanReac AppliChem) and goat anti-rabbit IgG Alexa 647 secondary antibody (1000x, A21245, Invitrogen-ThermoFisher Scientific) diluted in the 1% BSA blocking solution for 1 h at room temperature. Images were taken with an Opera Phenix High-Content Analysis microscope (Perkin Elmer, Waltham, MA, USA) using 40X water immersion objective with 1.1 numerical aperture in confocal mode (DAPI: ex 405 nm; em 435–480 nm and Alexa 647: ex 640 nm; em 650–760 nm). The LC3B spots were counted in each cell, as follows. First, the cells were detected based on the DAPI staining with the “Find nuclei” software module (channel: DAPI, method: C, common threshold: 0.20, area: > 60 µm^2^, splitting coefficient: 9.0, individual threshold: 0.20, contrast: 0.15, output population: nuclei). By using “Filter image” (channel: Alexa 647, method: Smoothing, filter: Gaussian, width: 10 px, output image: smoothed 647) the background was decreased. The cell border was found with the “Find cytoplasm” menu (channel: Smoothed 647, method: F, membrane channel: Plane Map Alexa488, individual threshold: 0.05). “Select population” (population: nuclei, method: common filters with deletion the border objects, region: cell) and the cells were removed from the border. The spots were found with the “Find spots” (channel: Alexa647, ROI: cells (removed border objects) and cell, method: C, radius: ≤ 4.0 px, contrast: > 0.18, uncorrected spot to region intensity: > 1.7, distance: ≥ 1.0 px, spot peak radius: 0.5 px with calculate spot properties, output population: spots). Finally, spot intensity was calculated with the „Calculate intensity properties 1, 2 and 3″ (channel: Alexa 647, population: cells (removed border objects), region: spots [1], spot maxima [2] and spot borders [3], method: standard [1, 2, 3], quantile fraction 50%).

### HER2 immunostaining

HCS plates were pre-coated with JIMT-1 media for 1 h, and then, the JIMT-1 cells were plated in 75 µl (10 000/well). After 24 h, the cells were pre-treated with 20 µM sunitinib for 1 h which was then diluted to 10 µM by the addition of JIMT-1 medium containing 10 µg/ml Alexa Fluor647-conjugated trastuzumab and were incubated further for 3 h. Afterward, cells were washed twice with 1 × PBS and fixed (4% formaldehyde, 10 min, room temperature). Permeabilization was performed with 100% methanol (10 min, -20 °C), and then, cells were washed twice with 1 × PBS. 1% BSA was used for blocking (1 h, room temperature), while the primary mouse β-actin antibody (5000x, sc-47778) was diluted in 1% BSA with Triton-X 100 (333x) and incubated for 1 h at room temperature. After washing twice with 1 × PBS, the secondary antibody anti-mouse Alexa Fluor488 was diluted (1000x, A11001, Thermo Fisher Scientific) in 1% BSA with Triton X-100 (1 h, room temperature). Cells were washed twice with 1 × PBS and were stained with DAPI (1 µg/ml, 10 min, room temperature), and then, the excess was removed with washing steps. Images were taken with an Opera Phenix HCS microscope (20 × air objective with 0.4 numerical aperture in non-confocal mode). Intensity distribution of immunostaining was analyzed using Fiji (ImageJ).

### Statistical analysis

Data are presented as mean ± SEM of at least three independent experiments. Shapiro–Wilk test was used to analyze normality. Kruskal–Wallis test supplemented with Dunn’s post-hoc test was performed if distribution of data was not normal. If the data distribution was normal, one-way ANOVA complemented by Sidak’s or Dunnett’s post-hoc test or two-way ANOVA supplemented with Tukey post-hoc test were used. A *p* < *0.05* was considered as significant. Statistical analysis was performed with GraphPad Prism 8.0.1 (GraphPad Software Inc., San Diego, CA, USA).

## Results

### Setting up an HCS-compatible assay for the monitoring of ADCC

**Fig. 1:**
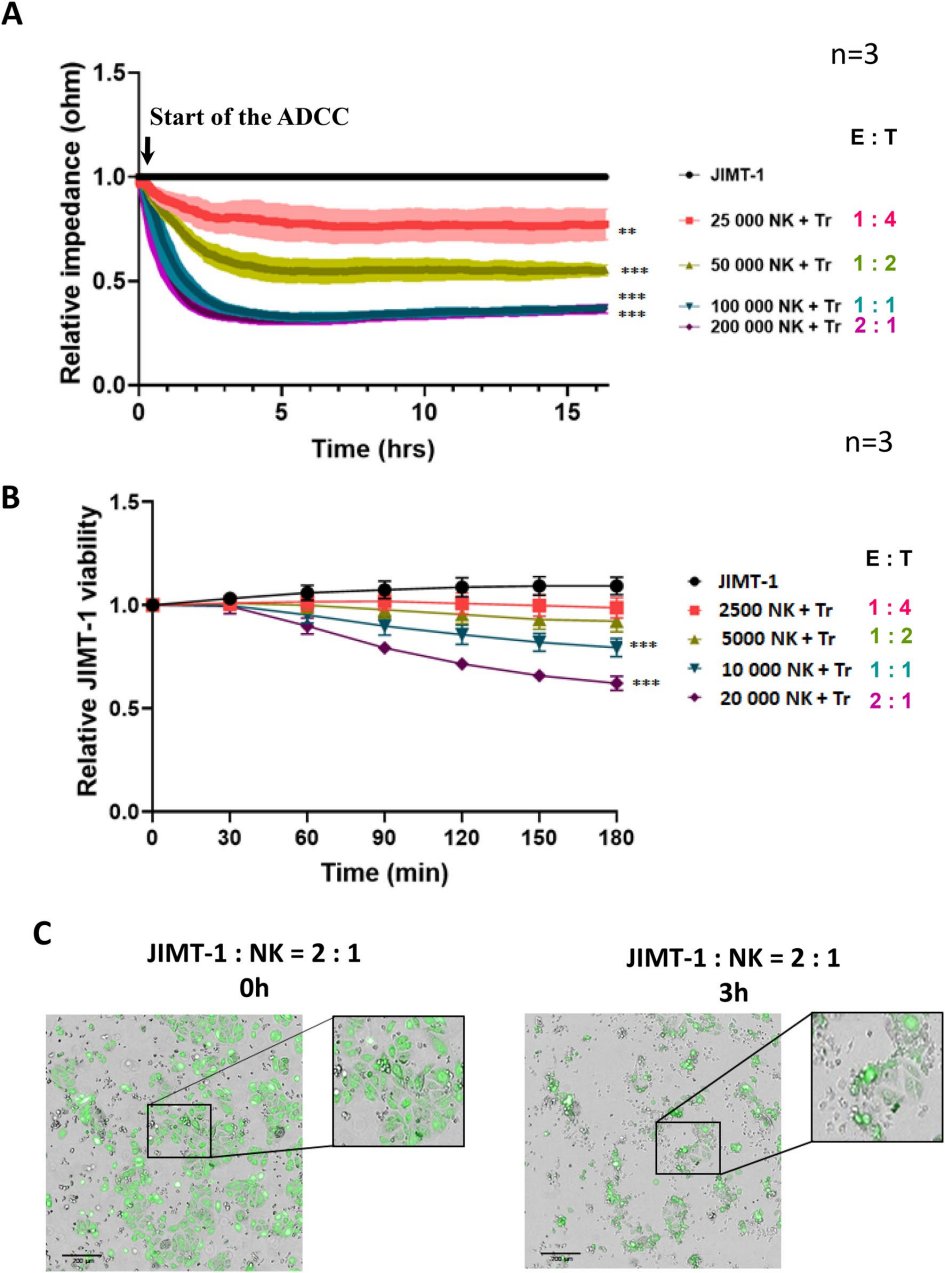
Optimalization of the ADCC assay**. a** JIMT-1 target cells were incubated with or without CD16.176 V.NK-92 cells at 1:4, 1:2, 1:1 and 2:1 effector target (E:T) ratio in either the presence or absence of trastuzumab (Tr). As a measure of cell viability, the impedance of wells was measured with ECIS. The effector cells were added to the JIMT-1 cells *(start of the ADCC)* when the impedance reached the plateau phase (~ 24 h). The diagram shows the average of 3 independent experiments (± SEM). Statistical analysis was calculated with two-way ANOVA followed by Tukey’s test. **b** JIMT cell viability was also determined at 3 h with the HCA-based calcein-AM assay using the same E:T ratios (1:4, 1:2, 1:1 and 2:1). Data were analyzed using two-way ANOVA and Tukey’s post-hoc test. **c** Representative pictures of calcein-stained JIMT-1 target cells during the ADCC (0 h and 3 h timepoints; E:T = 2:1). (**p < 0.01, ***p < *0.001*)

Our first goal was to set up a model for the quantitation of NK-cell-mediated ADCC. We chose the previously published setup that involves the CD16.176 V.NK-92 natural killer cell line and the HER2 positive JIMT-1 breast cancer cells. This cancer cell line was selected as it is known to be resistant to trastuzumab thus representing a clinically challenging cellular model. ECIS proved to be superior to some alternative methods for the quantitation of ADCC in this model [14]. Therefore, the first optimization assays were run on the ECIS platform (Fig. 1a). Our data showed that the effects of different E:T ratios could be convincingly demonstrated with ECIS in a time-dependent manner. Based on these data, we concluded that in subsequent experiments, we should focus on the first 3-h time window. Although the ECIS is a sensitive and reliable technology for the measurement of ADCC, it is not suitable for high-throughput screening. Therefore, we began to adapt the ADCC assay to an HTS-compatible platform (Fig. 1b). Previously, we successfully applied an image-based high-content analysis platform for the screening of potentially cytoprotective compounds [15]. Therefore, we tried a similar approach for the detection of JIMT-1 cell death in ADCC. Using a method based on the visualization of live cells with calcein-AM staining, we could detect cell death in a 3 h ADCC assay (Fig. 1b, c).

### High-content screening for ADCC modulators

**Fig. 2:**
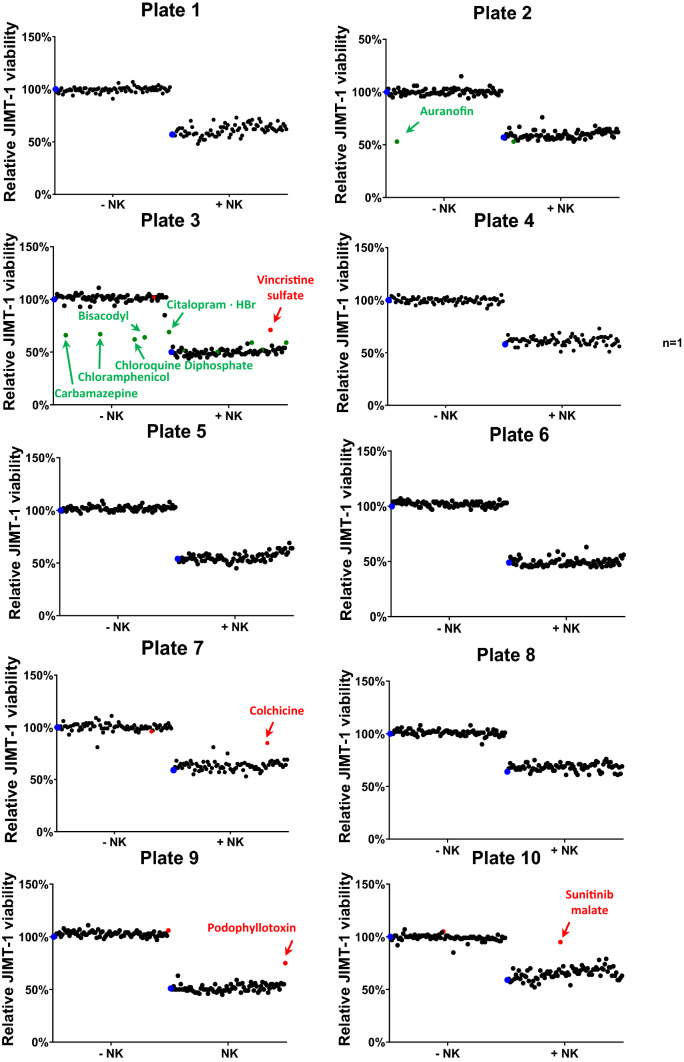
Identification of ADCC modulator compounds with HCS**.** Calcein-stained JIMT-1 cells were incubated with the compound library for 60 min in duplicates. In turn, half of the wells received unstained NK cells (E:T = 2:1) and trastuzumab (+ NK group), while the other half (-NK group) was used to assess the toxicity of the compounds on the target cells. Blue dots mark control samples in both sets. The stained target cells were counted immediately after addition of NK cells and 3 h after co-culture. JIMT-1 cell survival was measured and analyzed with the HCA assay system as detailed in the Materials and methods section. Compounds that were toxic to JIMT-1 cells (at a 20% threshold) are shown in green. Red dots mark ADCC inhibitors (at least 20% decrease in ADCC efficiency)

**Fig. 3:**
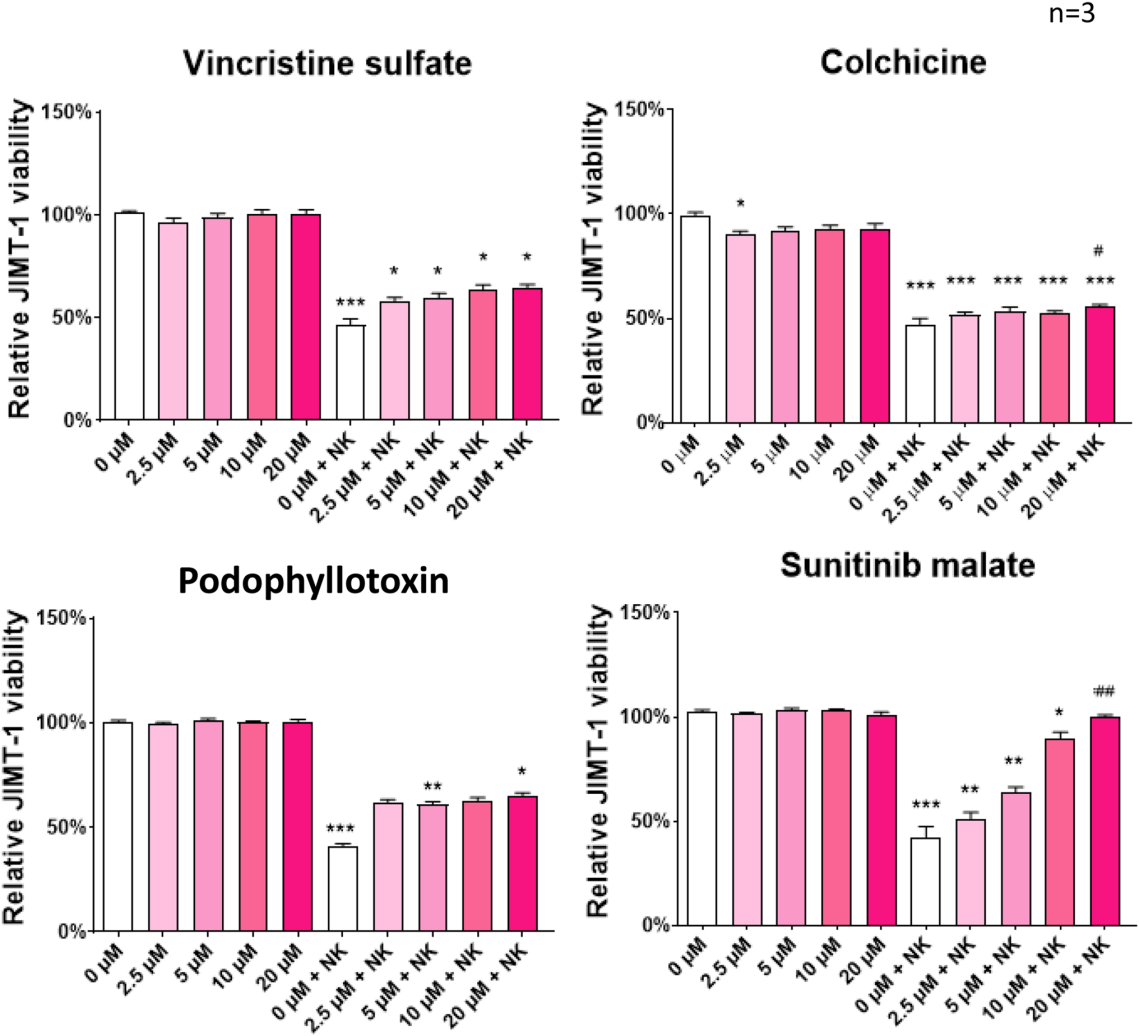
Quantification of the effect of hit compounds on ADCC efficiency. The calcein-stained JIMT-1 cells were pre-treated with the test compounds for 60 min followed by the addition of NK cells and trastuzumab. Cell viability was measured with the high-content analysis method. JIMT-1 viability is reported as an average (± SEM) of *n* = 3 independent experiments. The ADCC efficiency was determined with Kruskal–Wallis test and Dunn’s post-hoc test (for vincristine, podofilox and sunitinib) or one-way ANOVA and Sidak’s post-hoc test (for colchicine). (*p < 0.05, **p < 0.01, ***p < *0.001*)

Next, we performed a screen with a compound library containing 774 FDA approved drugs (Fig. 2). Addition of NK cells resulted in significant target cell death (56% as average of all 10 test plates). Six compounds caused higher than 20% toxicity in the target cells (in the absence of NK cells). Moreover, none of the test compounds enhanced ADCC using a threshold value of 20% higher target cell death compared to vehicle. However, four compounds (vincristine, colchicine, podophyllotoxin and sunitinib) caused more than 20% protection from ADCC (Fig. 2). We then retested these hit compounds and confirmed their ADCC inhibitory effects (Fig. S2 and Fig. 3). Fluorescent images of calcein-AM stained JIMT-1 cells clearly showed marked decrease in the number of viable (green) cells in the presence of NK cells and trastuzumab (Fig. S2). Pretreatment of cells with all four hit compounds provided protection from cell death (Fig. S2). Cellometric analysis of the fluorescent images confirmed this finding (Fig. 3).

**Fig. 4:**
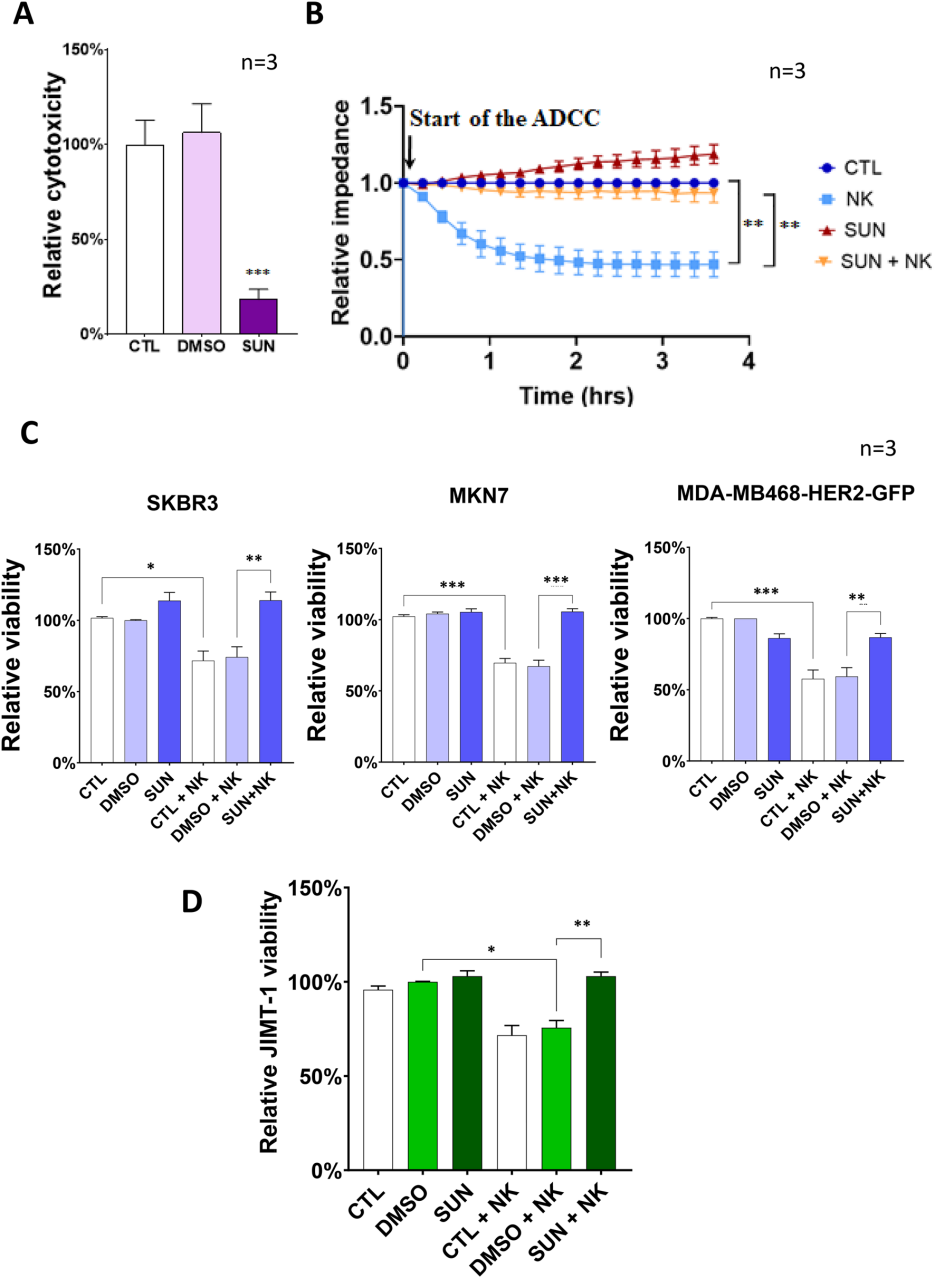
Confirmation of the inhibitory effect of sunitinib on ADCC. **a** JIMT-1 target cells were pre-treated with sunitinib for 60 min followed by the addition of NK cells and trastuzumab. Cell death was measured with the LDH release assay and cytotoxicity is reported as mean ± SEM of three independent experiments. Data were analyzed with Kruskal–Wallis test and Dunn’s post-hoc test. **b** JIMT-1 cells were allowed to reach the plateau phase in ECIS chambers (~ 24 h) and were then pre-treated with sunitinib for 1 h and treated with NK cells and trastuzumab as in “A”. Impedance data were analyzed with two-way ANOVA followed by Tukey’s test. **c** Calcein-stained SKBR3, MKN7 and MDA-MB468-HER2-GFP cells were pre-treated with sunitinib for 60 min followed by the addition of NK cells and trastuzumab. Target cell viability was measured with the high-content analysis method. Viability is reported as the average (± SEM) of *n* = 3 independent experiments. Statistical analysis was carried out with Kruskal–Wallis test and Dunn’s post-hoc test (for SKBR3 cells) or one-way ANOVA and Sidak’s post-hoc test (for MKN7 and MDA cells). **d** Calcein-stained JIMT-1 cells were co-cultured with NK cells after the pre-treatment with sunitinib then ADCC was performed in the presence of cetuximab (anti-EGFR monoclonal antibody) for 3 h. JIMT-1 viability was determined with the screening method and the statistical analysis was performed with Kruskal–Wallis test and Dunn’s post-hoc test. (*p < 0.05, **p < 0.01, ***p < *0.001)*

*Sunitinib inhibits ADCC in multiple HER2* + *cancer cell lines* Three of the hit compounds (vincristine, cholchicine and podophyllotoxin) can destabilize micrutubules, an effect known to inhibit NK cell function [16]. Therefore, we turned our attention to the multitargeted tyrosine kinase inhibitor sunitinib and aimed to characterize its ADCC inhibitory effect. The inhibitory effect of sunitinib on ADCC was clearly visible in time-lapse videos recordings of ADCC cultures (supplementary videos S3 and S4). We tested sunitinib in two additional cytotoxicity assays. Both the LDH release assay and the gold standard ECIS method proved that sunitinib treatment confers resistance to JIMT-1 cells in ADCC (Fig. 4a, b). To show that the effect of sunitinib is not limited to a single target cell line and to a single antibody, we confirmed the ADCC inhibitory effect of the drug on three additional HER2 positive cancer cell lines (Fig. 4c) and in assays in which trastuzumab was replaced with the anti-EGFR antibody cetuximab (Fig. 4d).

### Sunitinib inhibits NK cell activation and alters JIMT-1 phenotype

**Fig. 5:**
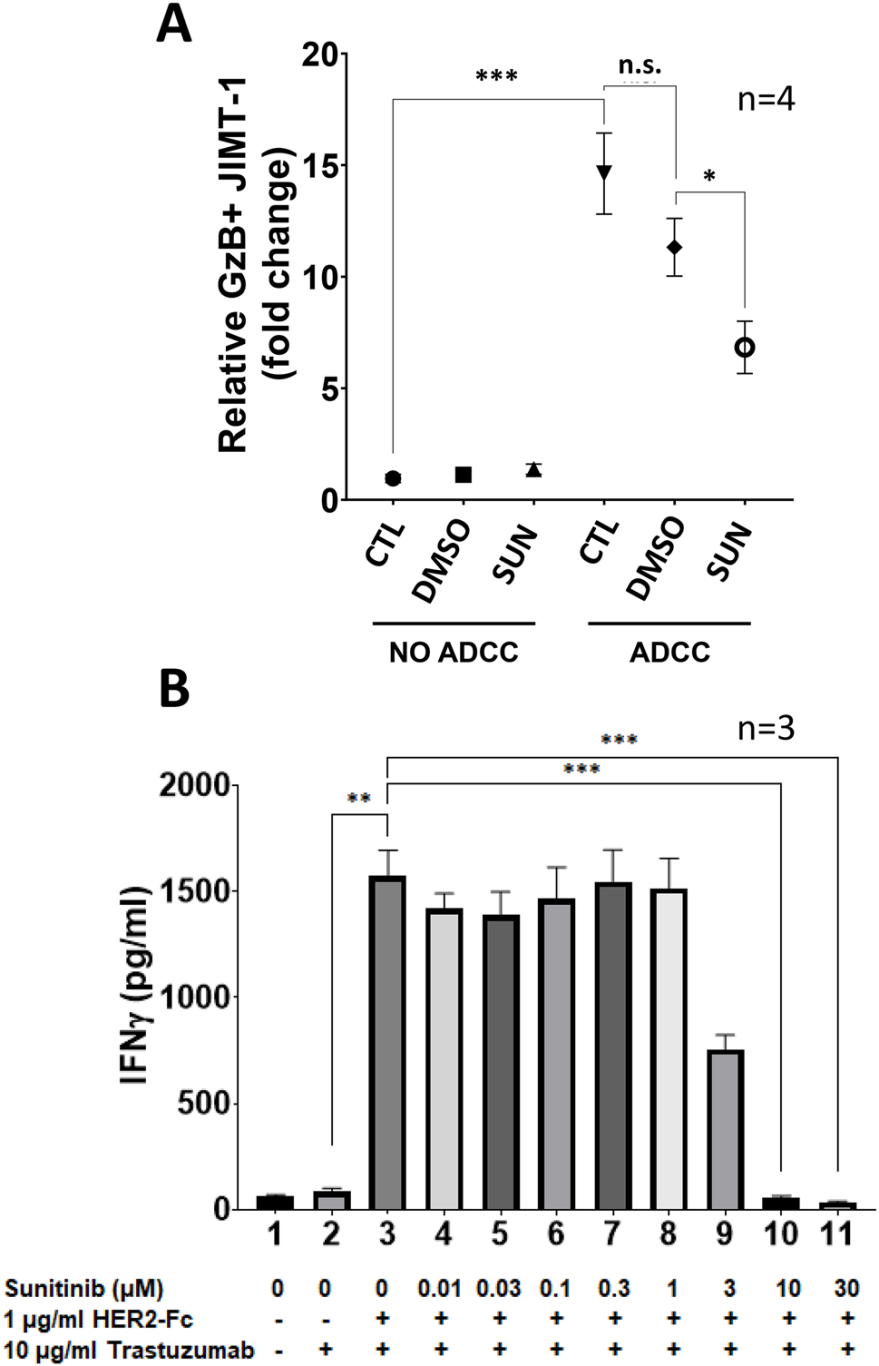
Sunitinib inhibits granzyme B release and interferon-γ production by natural killer cells. **a** Granzyme B (GzB) release from the effector NK cells was measured with flow cytometry in 3 h ADCC assays. The graph shows mean of 4 experiments (± SEM). Statistical evaluation was performed with one-way ANOVA and Sidak’s post-hoc test. **b** 2 × 10^5^ CD16.176 V.NK-92 cells ± 10 μg/ml trastuzumab were incubated with 1 μg/ml HER2-Fc protein in the presence of 0, 0.01, 0.03, 0.1, 0.3, 1, 3, 10 and 30 µM Sunitinib. After 24 h, IFNγ was determined in the culture supernatant by ELISA (*n* = 3; assay was performed in duplicates; histograms show mean ± SEM). Statistics were calculated with Kruskal–Wallis test and Dunn’s post-hoc test. (*p < 0.05, **p < 0.01, ***p < *0.001*)

**Fig. 6:**
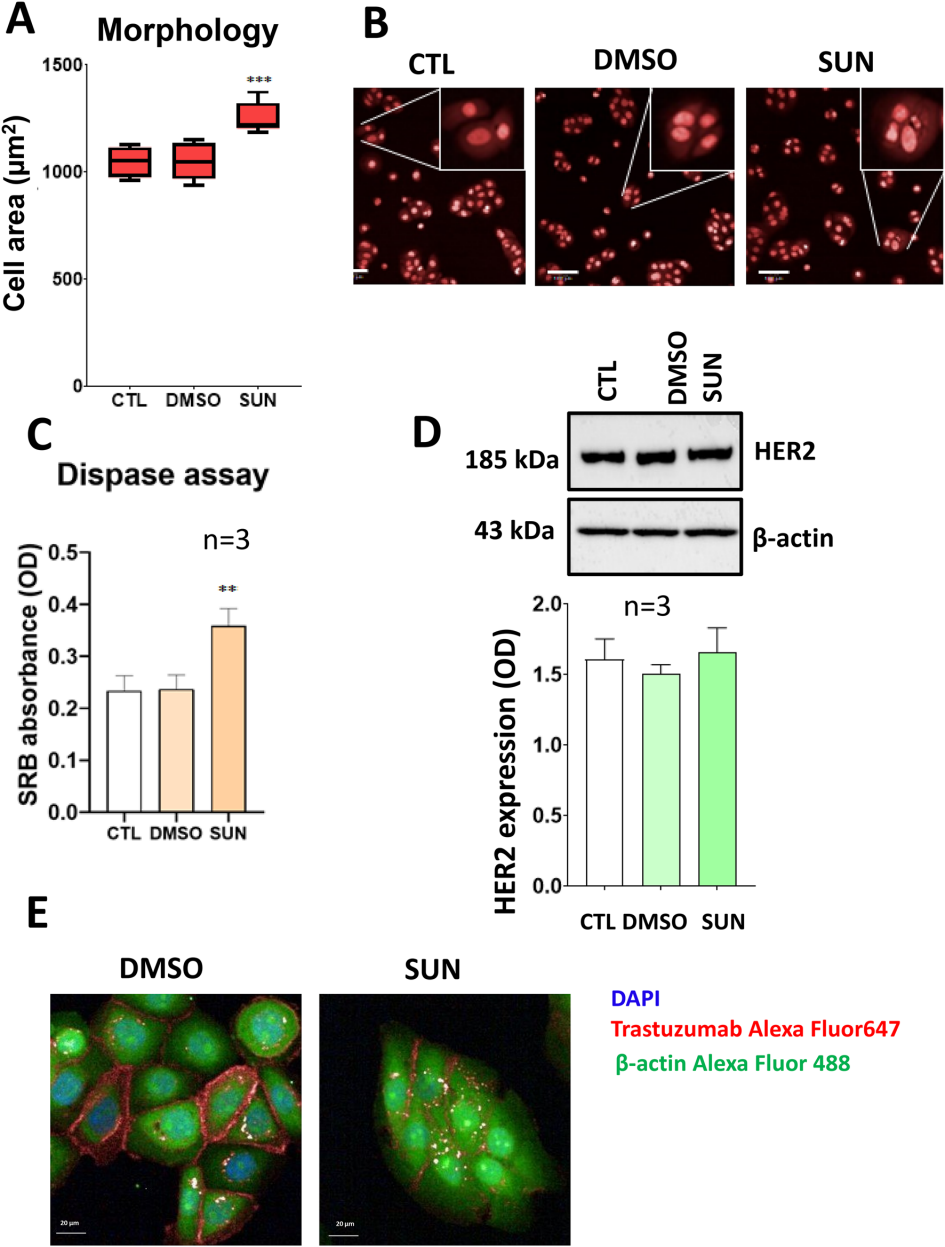
Effects of sunitinib on JIMT-1 cells. **a** JIMT-1 cells were treated with sunitinib for 4 h, stained with DRAQ5 and cell morphology was analyzed with the high-content analysis method. Median of the cell area is presented (± SEM). The diagram shows 3 independent experiments. Data were analyzed with one-way ANOVA and Sidak’s test. **b** The pictures were taken with a 20 × objective in confocal mode and representative images are shown. Scale bar is 100 µm. **c** JIMT-1 cells were treated with sunitinib for 4 h and adherence strength was determined with dispase assay as described in the Materials and methods section. Statistical evaluation was carried out with one-way ANOVA and Sidak’s post-hoc test. **d** JIMT-1 cells (300,000) were treated with sunitinib for 4 h and HER2 was detected with Western blotting. Sunitinib did not change the amount of HER2. Statistical analysis of densitometric values was carried out with one-way ANOVA followed by Sidak’s post-hoc test. **e** JIMT-1 cells (10,000) were pre-treated with sunitinib for 1 h, and then, cells were stained with Alexa Fluor 647 conjugated trastuzumab (red anti-HER2 antibody in JIMT-1 media) for 3 h. The green color indicates β-actin, while the nuclei were stained with DAPI. The pictures were taken with the Opera Phenix microscope (20 × air objective with 0.4 numerical aperture in non-confocal mode)

One of the cytotoxic molecules of NK cells in ADCC is granzyme B. Therefore, we determined whether the granzyme B-mediated cytotoxic pathway was affected by sunitinib treatment. We found that granzyme B reaches JIMT-1 cells during ADCC and sunitinib reduced granzyme B positivity of the target cells (Fig. 5a). This finding suggested that NK cell activation may be compromised by sunitinib. Indeed, sunitinib inhibited interferon-γ production by trastuzumab + HER2-Fc stimulated NK cells (Fig. 5b). Moreover, we were also looking for signs of an altered target cell phenotype as a potential underlying mechanism of cytoprotection in sunitinib-treated cells. Microscopic observation of sunitinib-treated cells indicated altered morphology (Fig. 6a). Morphometric analysis identified a notable increase in cell size (Fig. 6b). Furthermore, sunitinib-treated cells displayed stronger surface adherence as determined in dispase assays (Fig. 6c).

### Sunitinib induces downregulation of cell surface HER2 expression

The target molecule of trastuzumab-dependent NK cell-mediated ADCC is HER2. We hypothesized that sunitinib may interfere with the expression, membrane targeting or recycling of HER2. Immunofluorescent staining of HER2 revealed that JIMT-1 cells display heterogeneity in HER2 expression, but many cells express prominent membrane-associated HER2. Sunitinib had no effect on the total HER2 expression as indicated by Western blots (Fig. 6d) and cellometric analysis of HER2 immunostaining (data not shown) but markedly reduced cell surface expression of the receptor (Fig. 6e). Decreased membrane-associated HER2 may suppress cell proliferation. Indeed, cell cycle analysis indicated a partial G0/G1 arrest in sunitinib-treated cells (Suppl. Fig. S5) which may be due to insufficient growth factor signaling [17].

### Sunitinib induces autophagy

**Fig. 7:**
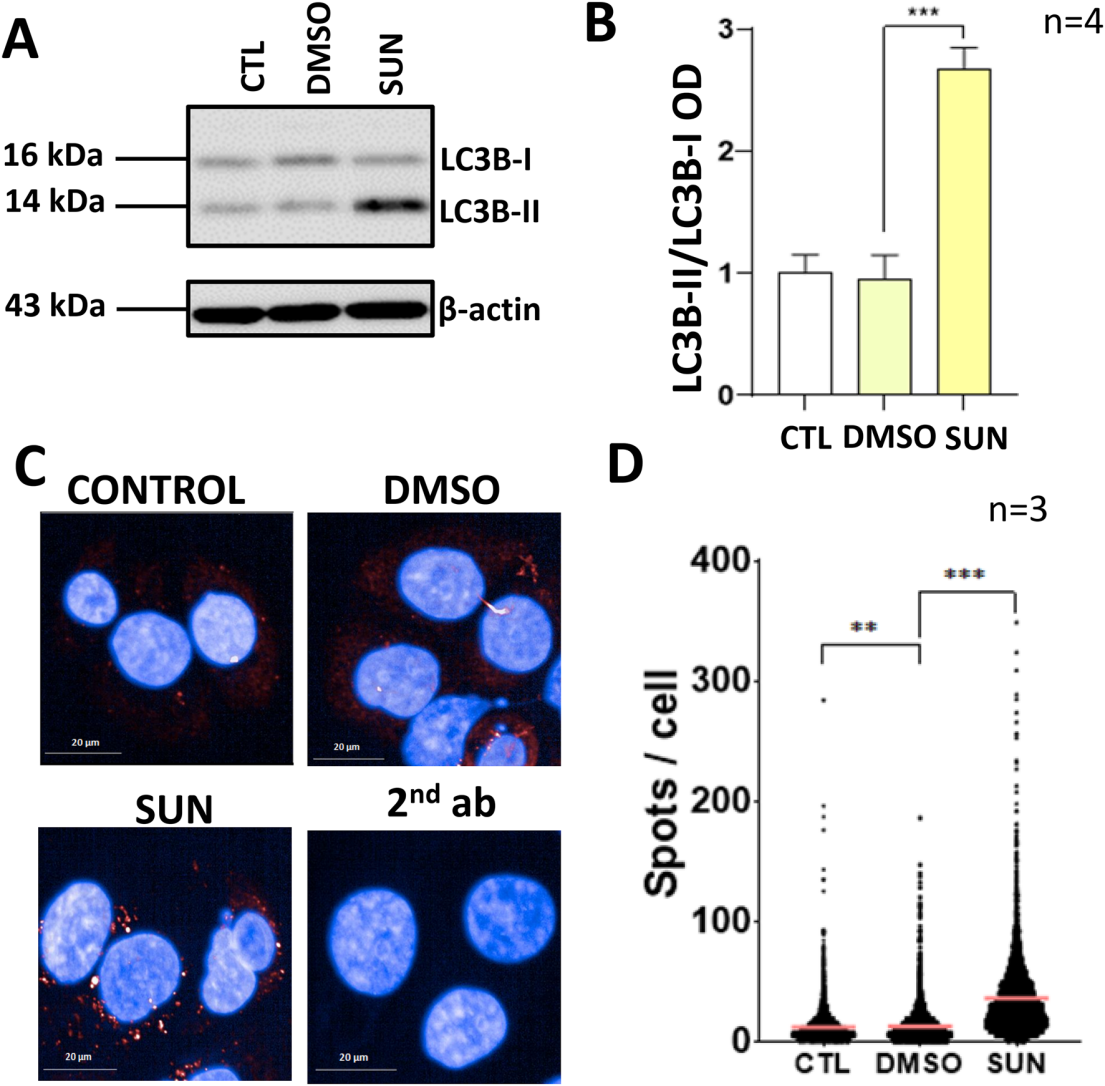
Sunitinib induces autophagy in JIMT-1 cells. **a** JIMT-1 cells (300,000) were treated with sunitinib for 4 h and LC3B was detected with Western blotting. Sunitinib increased the amount of LC3B. **b** Bands on the western blots were quantitated using densitometry. For the statistical analysis, one-way ANOVA was used with Sidak’s post-hoc test. **c** JIMT-1 cells were treated as in “A” and then stained for LC3B. Images were taken with Opera Phenix microscope (magnification was 40x). **d** Mean (pink line) LC3B protein expression per cell (± SEM) was calculated in 3 independent experiments. Minimum 3682 data points were used for the calculation. Kruskal–Wallis test and Dunn’s post-hoc test (**p < 0.01, ***p < *0.001)* were used for statistical evaluation

The autophagy pathway can provide protection against various cytotoxic stimuli [18–20]. Therefore, we investigated whether sunitinib induces autophagy in JIMT-1 cells. The autophagy marker LC3-I is a member of the ubiquitin-like LC3 protein family. Upon autophagy induction, LC3-I becomes lipidated with phosphatidyletanolamine. The lipid-modified variant is known as LC3-II. We have detected the autophagosome/autolysosome marker LC3-II by western blotting (Fig. 7a, b). In sunitinib-treated cells, the amount of LC3-II increased (Fig. 7a, b). The number of autophagosomes is indicated by the LC3 puncta. Immunofluorescent staining for LC3 revealed that autophagosome number increases in sunitinib-treated cells (Fig. 7c, d).

### Sunitinib also confers ADCC resistance to JIMT-1 cells in spheroids

**Fig. 8:**
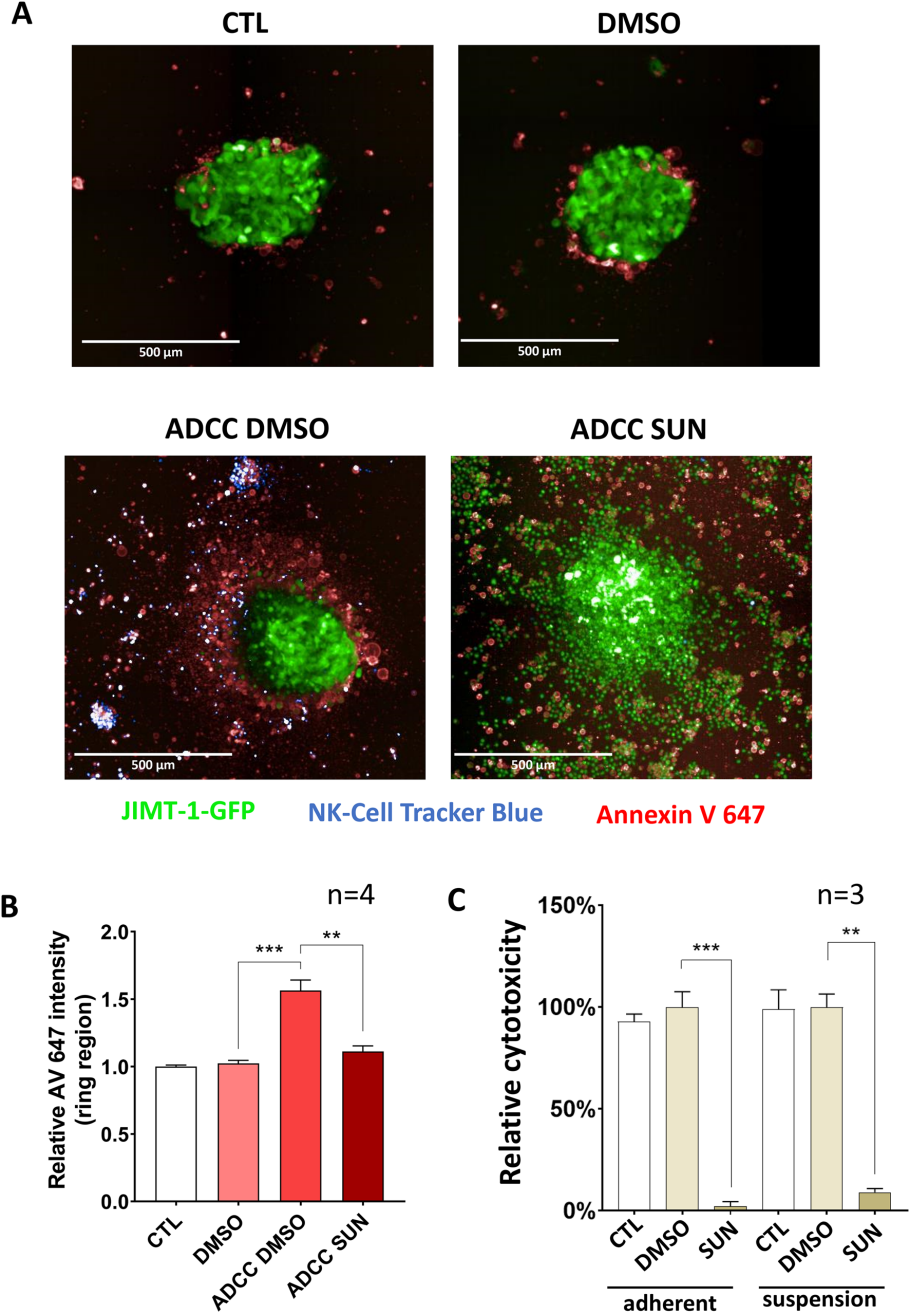
Sunitinib inhibits JIMT-1 cell apoptosis in a 3D ADCC model. **a** JIMT-1-GFP cells were used to generate spheroids. Trastuzumab (10 µg/ml) and NK92 CD16 cells (stained with Cell Tracker Blue) were added to spheroids (E:T ratio of 20:1) in the presence or the absence of sunitinib (20 μM). After 24 h, cells were stained with Annexin V 647 (red color) for 1 h. Representative images are shown in panel A. Images of 3 spheroids/treatment were taken and analyzed for the intensity of the ring region. **b** The columns represent the intensity of Annexin647 around the JIMT-1 spheroids. Means ± SEM of 4 independent experiments are shown. The statistics were calculated with Kruskal–Wallis test followed by Dunn’s post-hoc test. **c** Before the seeding of JIMT-1 cells, the wells were coated with Pluronic F-127 to prevent cell attachment. On the next day, JIMT-1 target cells were pre-treated with sunitinib for 60 min followed by the addition of NK cells and trastuzumab. Cell death was measured with the LDH release assay and cytotoxicity is reported as mean ± SEM of three independent experiments. Data were analyzed with Kruskal–Wallis test and Dunn’s post-hoc test. (**p < 0.01, ***p < *0.001*)

3D cultures more closely resemble in vivo situations than 2D cultures. Therefore, we generated GFP expressing JIMT-1 cells and grew spheroids from these cells. Addition of NK cells to these spheroids caused cell death in the JIMT-1 cells as indicated by Annexin V positivity (Fig. 8a, b). Apoptotic (Annexin V positive) cells emerged in the peripheral zone of the spheroids. Pretreatment of the spheroids with sunitinib reduced Annexin V staining in the spheroids (Fig. 8a, b). These data further confirmed the cytoprotective effect of sunitinib in the ADCC model. Signaling through integrins can provide survival signals in various models [21–24]. We compared cell death of adherent and non-adherent target cells in our model but found no difference between the two conditions (Fig. 8c).

## Discussion

NK cells play a central role in anti-tumor immunity. Their anticancer activity is regulated by a balance between activating or suppressive receptors. NK cells express Fcγ receptors through which they can bind tumor cell-bound antibodies such as trastuzumab. Bridging NK cells with cancer cells may result in cancer cell death even if the cancer is resistant to trastuzumab in vitro [6].

Harnessing NK cells for anti-cancer cell therapy or boosting their ADCC activity may be of great importance in the treatment of various forms of cancer. Moreover, combination therapies including those that involve immunotherapies have been extensively studied aiming to identify potential synergistic combinations [9]. Furthermore, understanding the effects of chemotherapeutics on the antitumor immune responses may help circumvent potential antagonistic effects.

Our HCS model proved suitable for the identification of ADCC modulator compounds. With the exception of sunitinib, all hit compounds including vincristine, colchicine and podophyllotoxin act by destabilizing microtubules [25]. This known effect likely explains their ADCC inhibitory actions as it has been previously demonstrated that key aspects of NK cell actions (e.g., migration, immune synapse formation and target cell killing) are supported by the cytoskeletal network dynamics [26]. In contrast to microtubular disrupting agents, sunitinib appeared to be a novel ADCC modulator. Therefore, in the second part of our study, we focused our attention on this anticancer agent.

Sunitinib had a concentration-dependent inhibitory effect on ADCC. The underlying mechanism of this effect is complex. The finding that sunitinib inhibits NK cell activation appears to be a key component of sunitinib’s action on ADCC. Decreased granzyme B in sunitinib-treated target cells indicates impaired delivery of the enzyme from the NK cell to the target. Impaired granzyme B trafficking in sunitinib-treated cultures may be due to suppressed NK cell activation as indicated by reduced IFNγ production in trastuzumab + HER2-Fc stimulated sunitinib-treated NK cells. The precise mechanism by which sunitinib impairs NK cell function in ADCC requires further investigation.

We also observed an altered morphology in sunitinib-treated target cells. To characterize this latter effect, we carried out morphometric analysis of the cells, and these experiments revealed that the area occupied by the sunitinib-treated cells was larger than that of control cells. We hypothesized that alteration in cell substrate adherence may lead to enhanced cell spreading which may be linked to ADCC resistance of sunitinib-treated cells. Our dispase assay data (Fig. 6c) showed that sunitinib induces stronger attachment of cells to the plate surface. The most likely mechanism involves upregulated expression of integrins. Signaling through adhesion molecules has been shown to result in resistance of cells to various cytotoxic agents. For example, in a model of oxidative stress-induced parthanatos, cell density-dependent signaling leads to downregulation of PARP1, the central mediator of parthanatos, conferring resistance against oxidative stress [21]. Integrin signaling converges on the prosurvival kinase Akt which has been linked to apoptosis resistance of adherent cells [27]. Dependence of epithelial cells on adherence is best exemplified by the anoikis cell death pathway triggered by detachment of cells from the extracellular matrix [28]. The contribution of integrin signaling to the ADCC resistance of sunitinib-treated cells requires further investigation. Lack of Akt activation (Suppl. Fig. S6) and lack of difference between ADCC sensitivity of adherent and non-adherent JIMT-1 cells suggest, however, that integrin signaling likely does not represent a central mechanism of sunitinib induced resistance in our model. Of note, altered cell morphology observed in sunitinib-treated cells may also indicate epithelial mesenchymal transition (EMT). In renal carcinoma cells, sunitinib induces EMT which has been linked to sunitinib resistance via EGFR activation [29]. Although we have not experimentally addressed this possibility in our current model, it cannot be excluded that sunitinib-induced ADCC resistance might also be due to EMT induction.

Another pathway we considered as a mechanism underlying the protective effect of sunitinib was autophagy induction. In a simplified view, autophagy is a recycling pathway triggered primarily by starvation [30]. By mobilization of the cells own proteins and lipids, autophagy may provide an escape mechanism from cell death when the primary cell fuel, glucose is scarce [30]. Our current view of autophagy is more complex and considers autophagy as a central pathway of cell homeostasis that also involves degradation and recycling of damaged or dysfunctional organelles or cell components. Even though autophagy can lead to cell death (autophagic cell death; [31]), under most conditions it acts as a cytoprotective mechanism. Blocking autophagy has been proposed to overcome resistance of cancer cells, including breast cancer cells, to chemotherapy [32]. In preclinical models, HER2 induced autophagy contributed to trastuzumab resistance of HER2 positive breast cancer cells [33]. Therefore, it has been proposed that autophagy inhibition might enhance the sensitivity of HER2 positive breast cancer to trastuzumab [32, 33]. Even though JIMT-1 cells express increased number of HER2, they are resistant to trastuzumab treatment [34]. However, they remain sensitive to trastuzumab-mediated ADCC [6]. Our current data suggest that autophagy may also suppress sensitivity of JIMT-1 cells to ADCC and thus autophagy induction may contribute to the desensitizing effect of sunitinib.

A key finding potentially underlying the ADCC protective role of sunitinib may be the downregulation of membrane-localized HER2. One scenario we investigated involved a possible inhibition of *her2* gene expression as reported in other studies [35, 36]. Trastuzumab itself may induce reduced *her2* gene expression but this effect depends on the engagement of the NK cell receptor [35]. Gene expression of HER2 is not likely to change in sunitinib-treated cells as total cellular HER2 level was unaltered. Thus, sunitinib may interfere with membrane-targeting of HER2. Mechanisms regulating membrane retention of HER2 are not well understood but are known to involve HER2 signaling and interaction of HER2 with Hsp90 [37]. How sunitinib perturbs membrane retention of HER2 requires further investigation.

In conclusion, we set up a cell morphometric assay for the quantification of trastuzumab-dependent ADCC. Screening of a small compound library yielded the multitargeted kinase inhibitor sunitinib as ADCC suppressing agent. Potential mechanisms underlying the anti-ADCC effect of sunitinib include inhibition of NK cell activation and decreased membrane retention of HER2, induction of autophagy and stimulation of integrin signaling in target cells. Experiments in which NK cells and target cells were exposed to the drug separately suggest that suppression of ADCC by sunitinib is mostly due to NK cell inactivation and effects on the target cells may be less important (Fig. S7). Nonetheless, the hierarchy of the multiple effects of sunitinib requires further investigation. Regarding the clinical significance of our study, our results raise caution about combination therapies using trastuzumab and sunitinib in trastuzumab-resistant breast cancer.

## Supplementary Information

Below is the link to the electronic supplementary material.

Supplementary file1 (PDF 626 kb)

Supplementary file2 (MP4 2883 kb)

Supplementary file3 (MP4 3042 kb)

Supplementary file4 (DOC 16 kb)

## Data Availability

The datasets generated during and/or analysed during the current study are available from the corresponding author on reasonable request.

## Declarations

### Human or animal participants

Additional declarations for articles in life science journals that report the results of studies involving humans and/or animals: not applicable.
