# Supplementary material for: The multitargeted receptor tyrosine kinase inhibitor sunitinib induces resistance of HER2 positive breast cancer cells to trastuzumab-mediated ADCC
Source: Cancer Immunol Immunother. 2022 Jan 23;71(9):2151–68. doi: 10.1007/s00262-022-03146-z (PMC9374626; doi:10.1007/s00262-022-03146-z)
Supplement: Supplementary file 1 — Supplementary file1 (PDF 626 kb) [file 262_2022_3146_MOESM1_ESM.pdf]

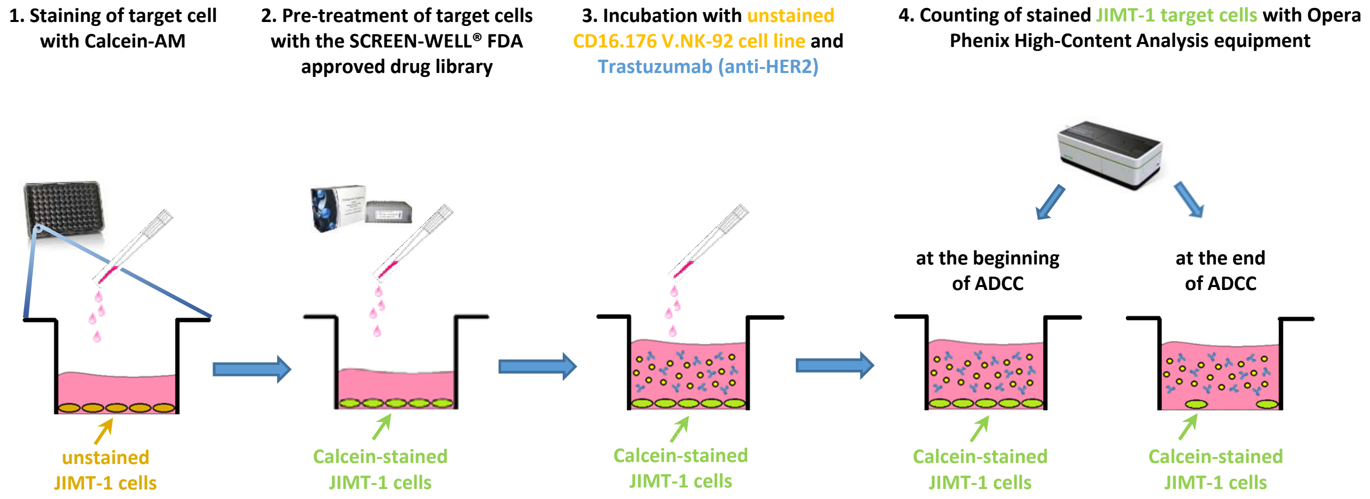

Supplementary Figure S1

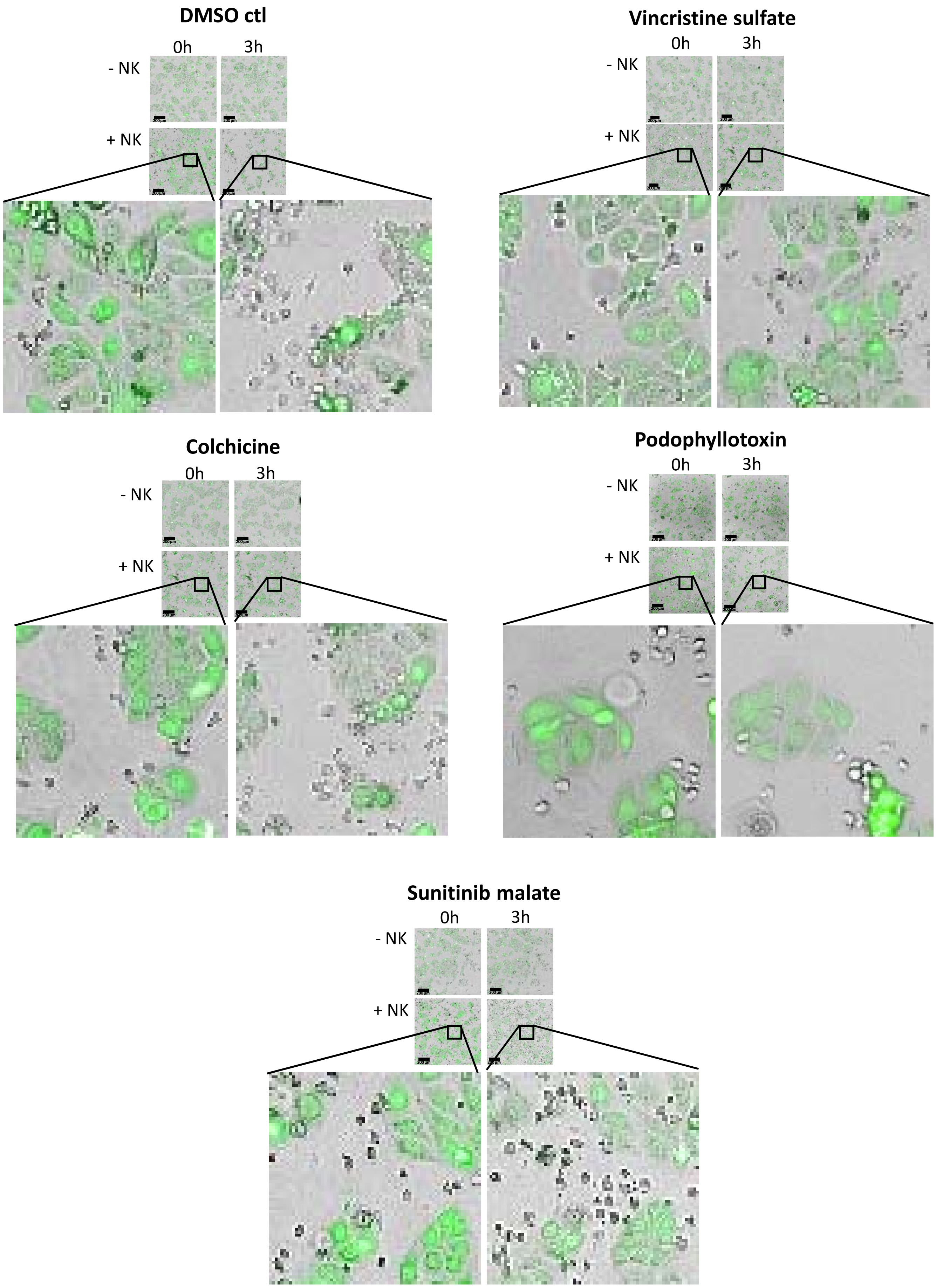

Supplementary Figure S2

Supplementary video S3: JIMT-1 cell killing in ADCC
https://www.dropbox.com/s/igp21zsykeyd440/ADCC.mp4?dl=0

Supplementary video S4: ADCC in the presence of sunitinib
https://www.dropbox.com/s/sjtsakoiwkzi4tb/sunitinib%20ADCC.mp4?dl=0

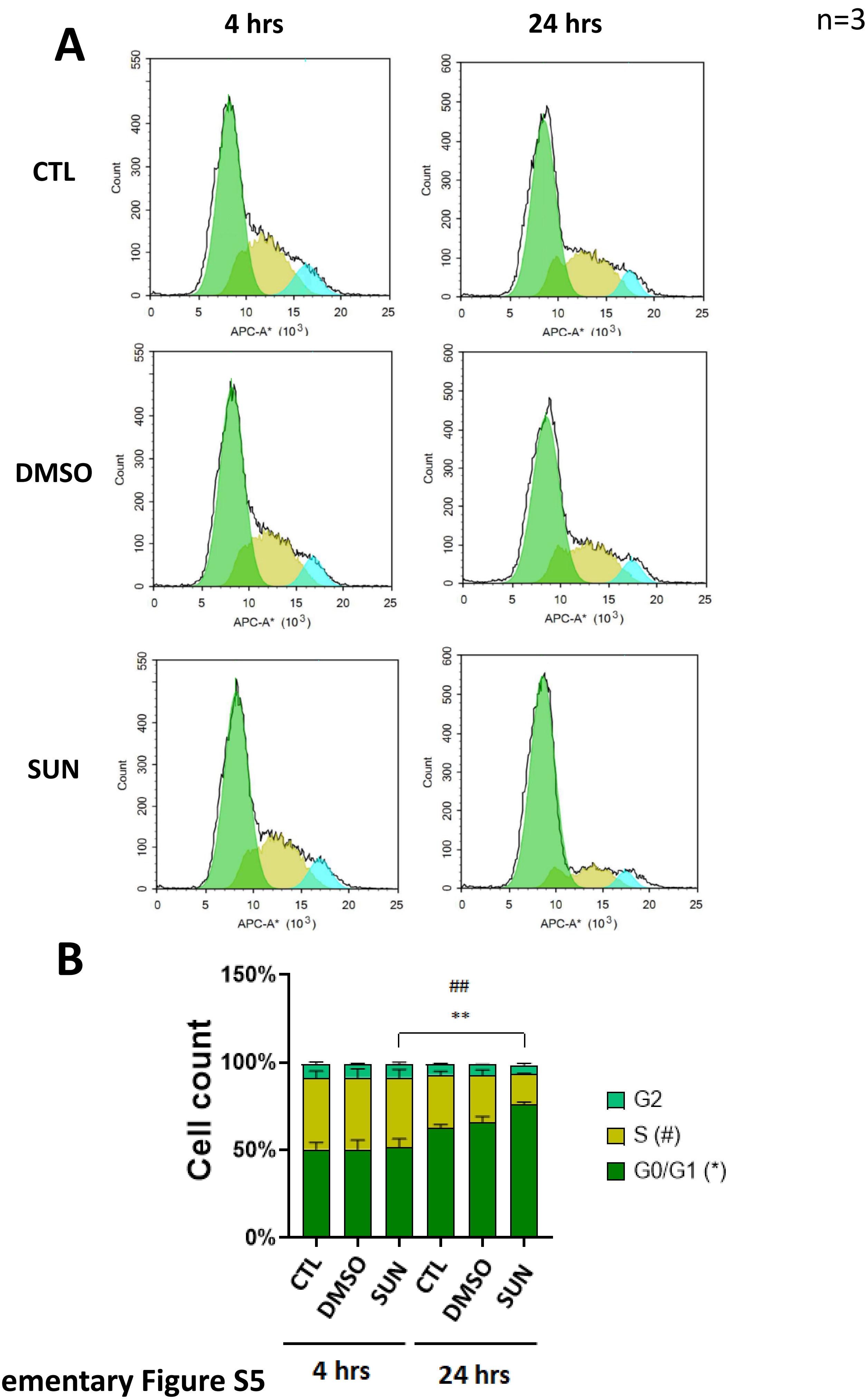

Supplementary Figure S5

n=3

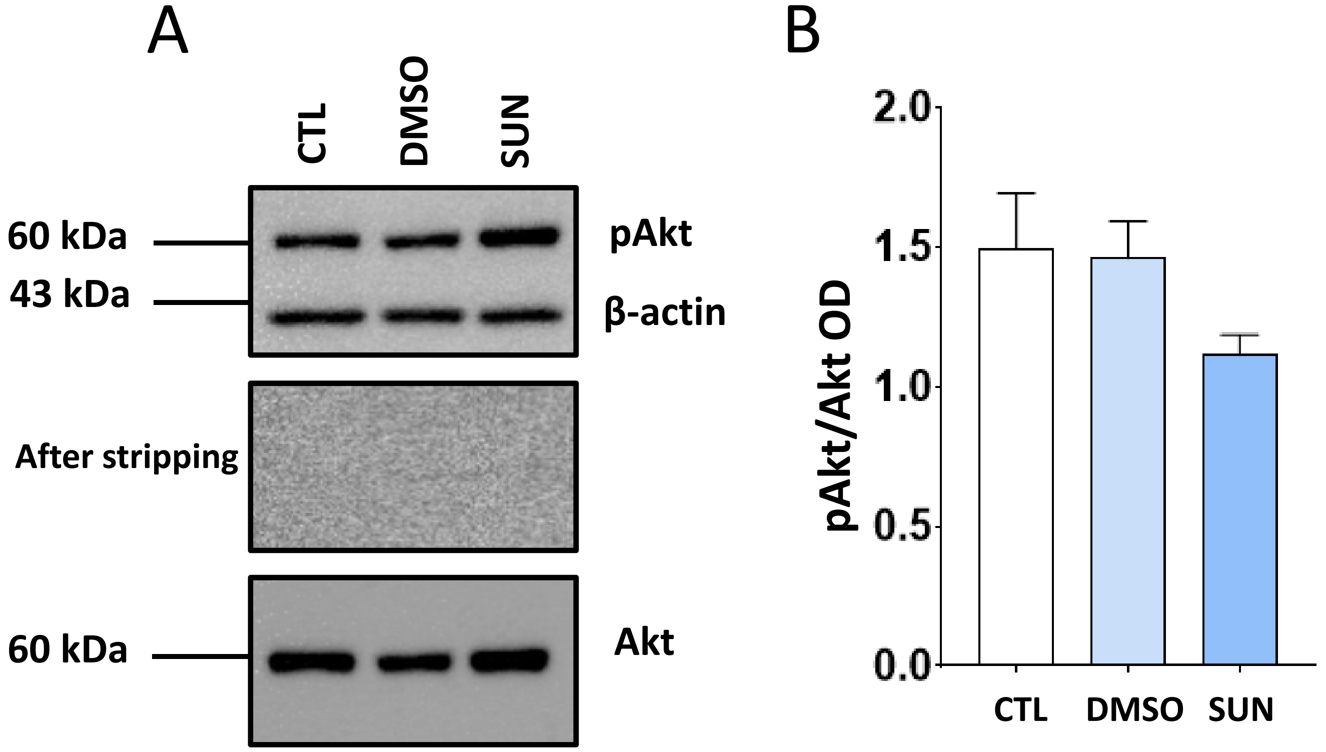

Supplementary Figure S6

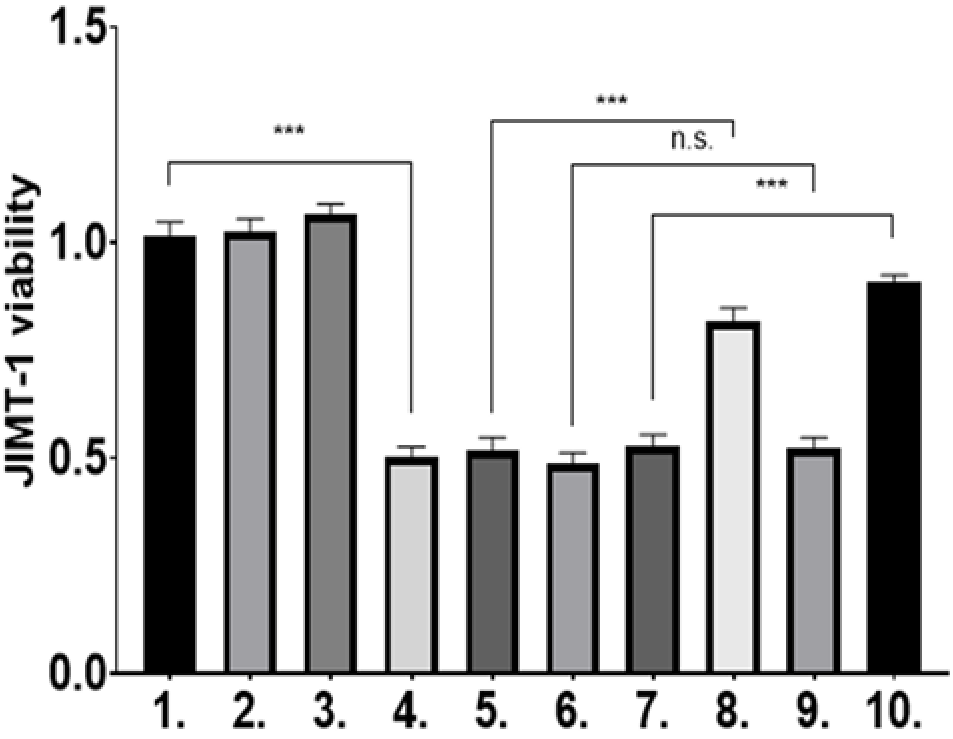

Pre-treatment 10 µM, 4 hrs	JIMT-1	-	dmso	sun	-	-	dmso	dmso	-	sun	sun
	NK	-			-	dmso	-	dmso	sun	-	sun

NO ADCC (1.–3.) ADCC (4.–10.)

Supplementary Figure S7
