# Supplementary material for: The multitargeted receptor tyrosine kinase inhibitor sunitinib induces resistance of HER2 positive breast cancer cells to trastuzumab-mediated ADCC
Source: Cancer Immunol Immunother. 2022 Jan 23;71(9):2151–68. doi: 10.1007/s00262-022-03146-z (PMC9374626; doi:10.1007/s00262-022-03146-z)
Supplement: Supplementary file 4 — Supplementary file4 (DOC 16 kb) [file 262_2022_3146_MOESM4_ESM.docx]

**Supplementary Figure S1. Compound library screening protocol**

**(1.)** JIMT-1 target cells were stained with 0.5 µM calcein-AM for 1 hour. **(2.)** Pre-treatment (1 h) of the cells with compounds of the drug library was performed with TECAN Freedom EVO 150 liquid handling robot. **(3.)** NK cells+trastuzumab (*ADCC*) or medium (*control*) was added. **(4.)** The number of calcein stained JIMT-1 cells was counted using an Opera Phenix High-Content Analysis equipment both at the beginning and at the end of the ADCC reaction (2:1=E:T ratio, 3-hour co-culture).

**Supplementary Figure S2. Confirmation of the ADCC inhibitory effect of the hit compounds**

ADCC was carried out with the hit compounds as described in figure 2 and images were taken at 0h and 3h. Representative images are shown. The pictures were taken with a 10x objective. Scale bars represent 200 µm.

**Supplementary video S3**

Calcein-stained JIMT-1 cells were co-cultered with unstained NK cells (E:T=2:1) and 10 µg/ml trastuzumab. Cultures were imaged with a 10x objective every 10 minutes for 3 hours to generate the timelapse video.

**Supplementary video S4**

Calcein-stained JIMT-1 cells were pretreated with sunitinib for 30 min and then co-cultered with unstained NK cells (E:T=2:1) and 10 µg/ml trastuzumab. FInal concentration of sunitinib was 10 µM. Cultures were imaged with a 10x objective every 10 minutes for 3 hours to generate the timelapse video.

**Supplementary Figure S5. Effect of sunitinib on the cell cycle**

**(A)** JIMT-1 cells were pre-treated with sunitinib for 4 or 24 h. The cells were collected and incubated with RNase A before staining with DRAQ5. The amount of DNA was analyzed by flow cytometry. **(B)** Quantitative analysis of the cell cycle. The graph shows the mean ± SEM of 3 independent experiments. Statistics were analyzed with two-way ANOVA and Tukey’s test. (***p<0.01)*

**Supplementary Figure S6. Sunitinib did not induce Akt phosphorylation**

**(A)** JIMT-1 cells (300,000) were treated with sunitinib for 4 h. pAkt and then, after stripping the membrane, Akt were detected with Western blotting. Sunitinib did not change the amount of neither Akt nor pAkt. **(B)** For the statistical analysis one-way ANOVA was used with Sidak’s post-hoc test.

**Supplementary Figure S7. Sunitinib reduced the killing ability of NK effector cells**

The calcein-stained JIMT-1 cells were pre-treated with sunitinib for 4 h followed by washing steps then the ADCC was started by addition of NK cells and trastuzumab. Alternatively, NK cells were pretreated with sunitinib for 4 h, then washed and added to JIMT-1 cells with trastuzumab. Cell viability was measured with the high-content analysis method. JIMT-1 viability is reported as an average (±SEM) of n=4 independent experiments. Statistics was calculated with one-way ANOVA and Sidak’s post-hoc test. (****p<0.001*)
